# The Effectiveness of Lifestyle Interventions for Older Adults According to Publicly Available Data

**DOI:** 10.1093/geroni/igaf122.2113

**Published:** 2025-12-31

**Authors:** Kevin Pritchard, Pureum Jeon, Eun-Young Yoo, Ickpyo Hong

**Affiliations:** Brigham and Women’s Hospital, Boston, Massachusetts, United States; Yonsei University Mirae Campus, Wonju, Kangwon-do, Republic of Korea; Yonsei University College of Software and Digital Healthcare Convergence, Seoul, Republic of Korea; Yonsei University Mirae Campus, Wonju, Kangwon-do, Republic of Korea

## Abstract

We used publicly available data from the “Well Elderly 2 Study” to compare the effectiveness of a lifestyle intervention in improving mental and physical health. The original study’s intention-to-treat (ITT) analysis was criticized for its small treatment benefit, likely because low adherence diluted the treatment’s effect. We re-analyzed the data to estimate the benefit of treatment adherence. This retrospective analysis included 424 older adults aged 60-95 in the U.S., who were randomly assigned from 2004 to 2008 to an occupational therapist-led lifestyle intervention or a no-treatment control, with follow-up starting at treatment assignment. To evaluate the effect on SF-36v2, we pooled data from participants who crossed over treatment every six months during the four-year period and analyzed them using generalized estimating equations (GEE) with robust standard errors. An ITT analysis estimated the effect of treatment assignment regardless of adherence. An AST (As-Treated) analysis estimated the effect of treatment adherence, accounting for selection bias from treatment deviation by using inverse probability of censoring weights. The ITT analysis showed a significant improvement in the mental composite (B = 0.54, 95% CI [0.15, 0.93]) but no significant effect on the physical composite (B = 0.71, 95% CI [-0.02, 0.03]). In contrast, the AST analysis showed improvements in both the mental (B = 0.77, 95% CI [0.49, 1.04]) and physical (B = 2.28, 95% CI [2.48, 2.75]) composites. While ITT analysis risks exposure misclassification, AST analysis is susceptible to selection bias if treatment deviation is intentional. Presenting both estimates improves clarity in findings.

